# Three-Dimensional Analysis of the Swimming Behavior of *Daphnia magna* Exposed to Nanosized Titanium Dioxide

**DOI:** 10.1371/journal.pone.0080960

**Published:** 2013-11-18

**Authors:** Christian Noss, André Dabrunz, Ricki R. Rosenfeldt, Andreas Lorke, Ralf Schulz

**Affiliations:** Institute for Environmental Sciences, University of Koblenz-Landau, Landau, Germany; RMIT University, Australia

## Abstract

Due to their surface characteristics, nanosized titanium dioxide particles (nTiO_2_) tend to adhere to biological surfaces and we thus hypothesize that they may alter the swimming performance and behavior of motile aquatic organisms. However, no suitable approaches to address these impairments in swimming behavior as a result of nanoparticle exposure are available. Water fleas *Daphnia magna* exposed to 5 and 20 mg/L nTiO_2_ (61 nm; polydispersity index: 0.157 in 17.46 mg/L stock suspension) for 96 h showed a significantly (*p*<0.05) reduced growth rate compared to a 1-mg/L treatment and the control. Using three-dimensional video observations of swimming trajectories, we observed a treatment-dependent swarming of *D. magna* in the center of the test vessels during the initial phase of the exposure period. Ensemble mean swimming velocities increased with increasing body length of *D. magna*, but were significantly reduced in comparison to the control in all treatments after 96 h of exposure. Spectral analysis of swimming velocities revealed that high-frequency variance, which we consider as a measure of swimming activity, was significantly reduced in the 5- and 20-mg/L treatments. The results highlight the potential of detailed swimming analysis of *D. magna* for the evaluation of sub-lethal mechanical stress mechanisms resulting from biological surface coating and thus for evaluating the effects of nanoparticles in the aquatic environment.

## Introduction

Although colloidal particles of geochemical and biological origin are abundant in the aquatic environment [1], the increasing use of engineered nanosized particles in industrial applications and consumer products raise eco-toxicological concerns [2]. Unknown toxicological effects especially of engineered nanoparticles on organisms require comprehensive multi-disciplinary investigations [3]. Scheringer [4] questioned that identical methods for chemical risk assessments can be applied to nanoparticles, whose environmental properties strongly depend on compositions, size distributions and surface treatment. Nano-sized titanium dioxide particles (nTiO_2_) are of particular concern [5] because they are produced in large quantities and can be found in the aquatic environment [6].

Numerous toxicological studies with nanomaterials have been conducted using the water flea *Daphnia sp.* [7], which is a key ecological model organisms and of high relevance in many aquatic food webs [8]. The results of these eco-toxicological studies, however, are not consistent. While, e.g., Lovern and Klaper [9] reported mortalities (LC_50_) for *Daphnia magna* at 5.5 mg/L nTiO_2_ after 48 h. Heinlaan et al. [10] observed that even a concentration of 2^.^10^4 ^mg/L nTiO_2_ is not acutely toxic. Zhu et al. [11] found only minor immobilization and no significant mortality of *D. magna* after 48 h exposure to 50 and 100 mg/L nTiO_2_, but a higher toxicity when exposure time was extended to 72 h. Lazorchak et al. [12] highlighted the importance of prolonged acute exposure times > 48 h and Dabrunz et al. [13] found a substantially higher toxicity when exposing *D. magna* for 96 h. The reason of the increased toxicity of nTiO_2_ following an exposure ≥ 48 h is related to the inhibition of the second molting. Most importantly, the study by Dabrunz et al. [13] observed an adhesion of nTiO_2_ to the daphnids exoskeleton, which had been previously described already by Baun et al. [14]. Dabrunz et al. [13] hypothesized that this biological surface coating by nTiO_2_ may affect the swimming performance of the water flea. Swimming behavior is a frequently used endpoint in toxicity testing with daphnids, however, it has not yet been studied in detail during nanoparticle exposure, although the observed biological surface coating suggests a relevant effect.

Not only immobilization or mortality but also reduced growth or behavioral changes of organisms are of ecological relevance as sub-lethal endpoints [15], [16]. Baillieul and Blust [17] found the growth endpoint to be a viable indicator of sub-lethal effects resulting from contaminants based on measurement of physiological energetics (scope for growth). Billoir et al. [18] came to a similar conclusion concerning growth using dynamic energy budget in toxicology analyses. Further studies [11], [19], [20] reported about growth and molting reduction using nanoparticles, but only on basis of long-term (≥ 21 days) chronic tests. A common approach for testing immediate sub-lethal impacts of exposure to toxic substances on daphnids is to observe changes in swimming behavior [15], [21], [22]. To compensate sinking due to their negative buoyancy [23], *D. magna* have to swim upward frequently, resulting in typical hop and sink motion [24]. Dodson et al. [15] have demonstrated that the swimming velocity was the most useful endpoint for parameterization of differences in swimming behavior. Even among a wide variety of behavioral, biochemical and metabolic parameters, Duquesne and Küster [22] identified the swimming activity and particularly the mean swimming velocity as the most sensitive parameter. Baillieul and Scheunders [25] describe an image analysis method, which allows for measuring the average velocity of many simultaneously moving similar objects. Using this method, Baillieul and Blust [17] showed that Cd^2+^ exposure reduced the growth rate and the swimming velocity of *D. magna* independently. Although, video analyses provide detailed information on the motions of groups as well as of individuals, growth and behavior depend on a variety of biotic and abiotic factors [24], [26], [27], making eco-toxicological studies using behavioral responses rather challenging. For technical reasons, numerous studies used planar, i.e., two-dimensional observations [16], [17], [22], where daphnids were confined in flat chambers or flow-through cells and restricted in their normal behavior.

Here we provide a method to analyze the three-dimensional swimming behavior of *D. magna* exposed to different concentrations of nTiO_2_. A novel two-camera tracking system was developed for the behavioral analyses of multiple and simultaneously swimming organisms. We use spectral analysis of the swimming velocities to quantify swimming activity. The mechanical impact of nTiO_2_ on *D. magna* is analyzed by relating the observed swimming activity to size and growth rate of the organisms. This new approach should facilitate our mechanistic understanding of nanoparticle effects on aquatic organisms.

## Materials and Methods

### Husbandry and exposure

Fifty neonate (age <24 h) *D. magna* (clone V) were separated from a permanent culture which is kept in ASTM medium (composition provided in the legend of Figure S1 in the Supporting Information) [28] within climate controlled chambers at 20±1°C. Animals in the culture were fed with green algae *Desmodesmus* sp. on a daily basis. Test organisms were transferred to the test vessels (cubic glass tanks with 8 cm side length) 1 h before addition of nTiO_2_ and start of the observations during the experimental period. Night and day cycles were simulated using warm-white LED (3450 lux; 450–760 nm, no UV) panels at 12 h:12 h periods. Five groups were composed, each containing 10 individuals. Two groups remained as control groups (C, C*) while three treatment groups (*T*_1,5,20_) were exposed to 1, 5 and 20 mg/L nTiO_2_. Relative oxygen concentration measured in *C* and *T*_20_ was always 100±2%, electrical conductivity measured in *C* and *T*_5_ was 541±1 µS/cm and pH was 8.2±0.2 during the experimental period. Following procedures previously established [13] the animals were not fed during the experimental period. The nTiO_2_ product (P25, Evonik Aeroxide®) had a nominal primary particle size of 21 nm, and the surface area (Brunner–Emmett–Teller; BET) was approximately 50 m^2^/g. Size distributions in undiluted, monodisperse stock suspensions were determined by dynamic light scattering (Delsa™ Nano C, Beckman Coulter, Krefeld) to be 60.7±0.5 nm (polydispersity index: 0.157; D90: 113.5±3.0 nm). In the present study, we followed the analytical methods as described in Dabrunz et al. [13], with the only difference that we quantified 49Ti instead of 47Ti, since quantifying this mass did work slightly more accurate for P25. Briefly, titanium concentrations (mass 47, 17.46 mg/L) in the stock were verified by Quadropole ICP-MS (XSeries2, Thermo Fischer Scientific, Dreieich) equipped with a FAST autosampler (ESI, Thermo Fischer Scientific, Dreieich), a peek spray chamber (Thermo Fischer Scientific, Dreieich) and a robust Mira Mist peek nebuliser (Burgener, Berkshire). The instrument was run in the collision cell mode with 5 ml He/H2 cell gas in order to avoid polyatomic interferences (e.g., PO+ or SiOH+) [13]. As observed in previous studies [13], nTiO_2_ precipitated by forming larger agglomerates leading to a reduction of 50% of the initial suspended particle concentration within 30 h (Figure S1).

### Video recording

Swimming behavior of the daphnids was observed using video recordings at (*t_0_*), 24 h (*t_24_*), 48 h (*t_48_*), 72 h (*t_72_*) and 96 h (*t­_96_*) after application of nTiO_2_ in all treatments as well as in the control group. During each recording, daphnids of each group were observed through two orthogonal side-windows of the respective vessel. We used two synchronously triggered 2048×2048 pixel CCD-cameras (FloSense 4M, Dantec Dynamics, Skovlunde) at a frequency *f_s_* = 14.8 Hz and with a grey-scale depth of 8 bit. The usage of telecentric lenses (TC4M, Opto Eng., Mantova) allowed obtaining distortion- and divergence-free images of the entire test tank. Transformation of pixel coordinates to metric units was performed using a constant conversion factor of 19.5 pixel/mm obtained from calibration measurements using a metric target. Cartesian coordinates ***x*** **_d_** of *n* simultaneously swimming *Daphnia magna d* were identified in each recorded image pair using an assignment algorithm [29]. The algorithm was first applied to each of the two-dimensional coordinates obtained from the two cameras and a second time to estimate three-dimensional coordinates of the organisms by combining the two perspectives. All observations were performed during the light cycle of the photo period using the same illumination as during the husbandry. Before each recording we waited 15 min to avoid abnormal swimming behavior caused by light fluctuations during the transport of the test tank from the husbandry to the camera setup.

### Organism sizes

Body length (*L*) of each individual organism of the groups was estimated from the video observations for each sampling time. *L* (±50 µm) corresponds to the length from the apex of the head to the abdomen of the organisms. All daphnids were identified and *L* was estimated by visual inspection of the side views if the individual organisms, where the corresponding image of the second perspective shows the front view of the same individual. Relative growth rate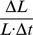 was estimated with time intervals Δ*t* = 24 h.

### Velocity analyses

Swimming behavior of all daphnids within the respective treatments was recorded for at least 3 min at *t*_0_, *t*_24_, *t*_48_, *t*_72_ and *t*_96_. Although the time of video recording relative to the application of nTiO_2_ were identical for each group, sampling of all groups took approximately 3 h. Each sampling started with the control group (*C*) and ended with a record of group *T*_20_. It has been shown that body size has a strong impact on the swimming performance of *Daphnia magna* [17]. Thus, a second control group (C*) was used and measured at the end of the 3 h interval to estimate the growth during the measurement phase. Swimming velocity components 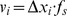, where 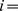 *v*, *h*_1_ and *h*_2_ denotes vertical or horizontal components, were calculated from corresponding displacements Δ*x_i_* of individual daphnids observed in subsequent images obtained at the frame rate *f_s_*. Two-dimensional observations from the individual cameras allow for analyzing magnitudes of swimming velocities 
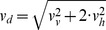
 along continuously recorded pathways of individual daphnids. The swimming velocities were averaged by calculating the temporal mean swimming speed 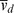 as well as the ensemble mean 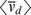 for each group. Three-dimensional coordinates and swimming speeds 
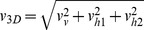
 allowed identifying predominant swimming directions and organisms located in the vicinity of the vessel walls. We noticed only minor differences 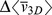 in ensemble mean swimming velocities estimated separately for daphnids swimming at distances 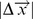 > 5 mm and ≤ 5 mm (about two body lengths) to the glass surfaces (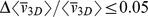). Horizontal velocity components were predominantly isotropic, i.e. 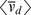 corresponds to 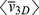 with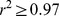, where *r* denotes the correlation coefficient. However, three-dimensional pathways were frequently fragmented into shorter segments and the corresponding velocities could not unambiguously assigned to distinct individuals. Hence, 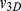 was only used for estimating ensemble mean sinking velocities using the criteria 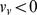, 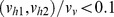 and 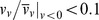 for passive sinking. Sinking velocities and corresponding body lengths had been used to estimate volume specific mass of the daphnids using Stokes law.

Swimming behavior was characterized using spectral analysis of the observed swimming velocities. Power spectral density *PSD* as a function of frequency *f* is estimated 
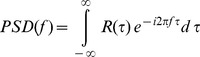
, where 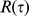 denotes the autocorrelation function of linearly de-trended 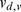, using Welch’s method [30], i.e. the Matlab (MathWorks, Natick) function psd( ) and method spectrum.welch, with a window length for Fourier transformation of 256 samples and 50% overlap. Variance in swimming velocity within a frequency range 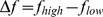
was estimated as 
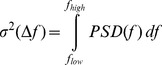
. Further the maximum of spectral variance (peak values) in the variance preserving spectra 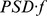 was estimated from 10-point moving averages of *PSD*.

### Statistics

Wilcoxon rank sum test was applied for testing significant differences in sizes, ensemble mean swimming velocity, mean peak values of variance preserving *PSD* and mean integral values of high frequency *PSD*. A two sample *t*-test was applied for testing significant difference of linear regression of two datasets, i.e. of the slopes of ensemble mean velocity versus length of the daphnids (see below). Differences are considered to be significant if *p*<0.05 for all tests, unless otherwise stated. Bonferoni correction was applied to identify significant differences affected by the problem of multiple comparisons.

## Results

### Growth rates and immobilization

Since the main aim of this study was to investigate the effects of nTiO_2_ concentrations on the swimming behavior of *D. magna,* we do not take immobilized organisms into account in our study. In accordance with Baillieul & Blust [17], we did not mix observations of mobile animals with that of immobilized animals. Hence only nine individuals constitute *T*_1_ at *t* > *t*_0_ and *T*_20_ at *t* > *t*_24_ and only eight individuals constitute *T*_5_ and *T*_20_ at *t* > *t*_48_.

**Figure 1:**
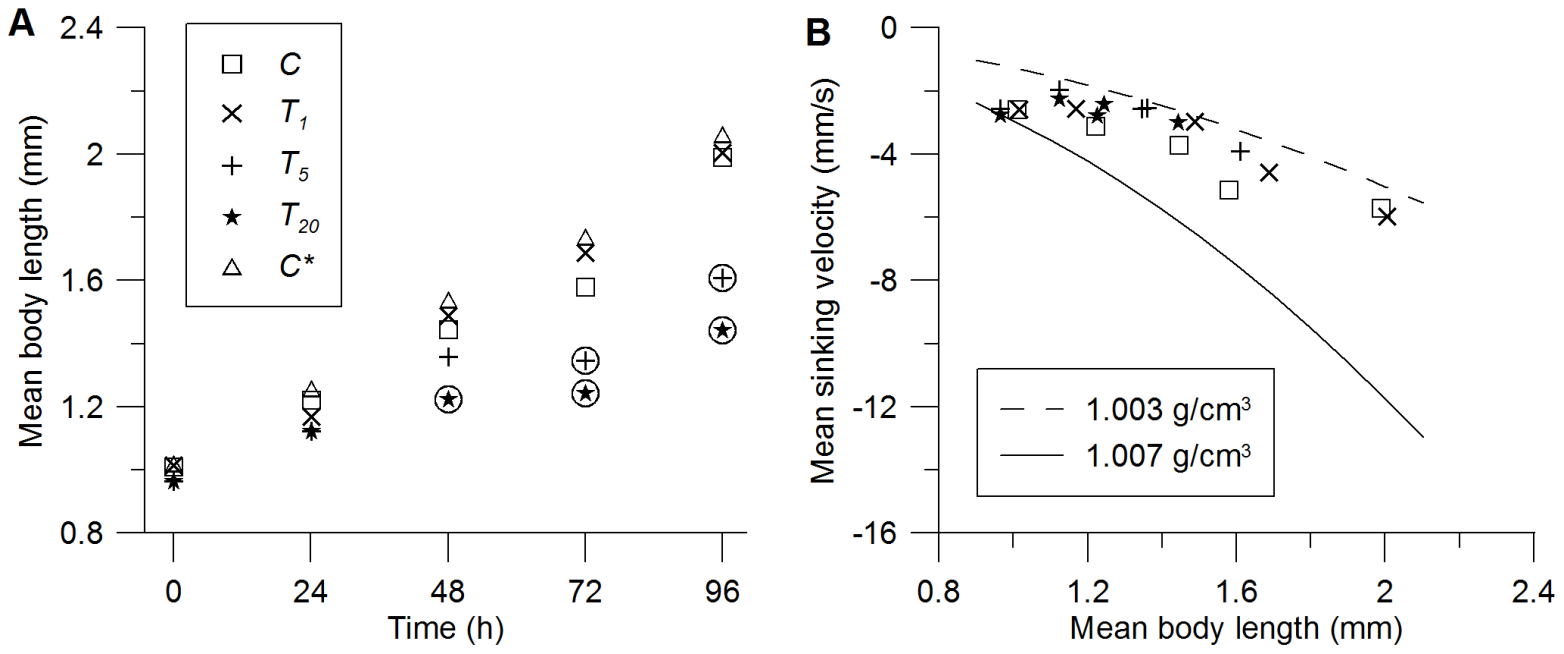
Mean body length and mean sinking velocity of *Daphnia magna.* (A) Body length in control group (*C*), treatment with 1 mg/L nTiO_2_ (*T*_1_), treatment with 5 mg/L nTiO_2_ (*T*_5_) and treatment with 20 mg/L nTiO_2_ (*T*_20_). Please note that ‘Time’ denotes the time of exposure and *C* (first record) and a second control C* (last record) cover each record period (approx. 3 h). Circles around the symbols denote significant differences (*p*<0.05) to *C*. Error bars are omitted for the sake of clarity. Please refer to Table S1 for standard deviations. (B) Mean sinking velocity vs. mean *Daphnia* body length and sinking veloicties estimated using Stokes’ law using organisms densities of 1.007 g/cm^3^ (black line) and 1.003 g/cm^3^ (grey line), respectively.

At *t*_0_, the initial length was 1.0±0.03 mm for all daphnids (Figure 1A; Table S1 in the Supporting Information). At *t*_96_ the control group *C* (2.0±0.15 mm) as well as *T*_1_ (2.0±0.16 mm) were significantly larger in comparison to *T*_5_ (1.6±0.26 mm, *p*  = 0.004) and T_20_ (1.4±0.29 mm, *p*  = 0.002). This difference translates to a reduced growth rate of –0.01 1/d for *T*_5_ and 0.02 1/d for *T*_20_ between *t*_48_ and *t*_72_. However, the growth rates estimated between other records of *T*_5,20_, i.e. from *t*_0_ to *t*_48_ and from *t*_72_ to *t*_96_, were 0.09…0.27 1/d. These growth rates were similar to the growth rates of *C* and *T*_1_ (0.09…0.26 1/d) during the entire experiment, i.e. from *t*_0_ to *t*_96_. The second control group C* measured at the end of the 3 h recording period deviated only 5 % (0.07±0.05 mm) from *C*. Thus, effects on the swimming behavior due to increased size of the organisms over the 3 h periods were considered negligible.

Absolute values of sinking velocities increased with increasing body length of *Daphnia magna* (Figure 1B). Corresponding volume specific mass was within 1.003 and 1.007 g/cm^3^. A decrease of approximately 3 mg/cm^3^ occurred mainly between *t­*_0_ and *t*_24_ in all treatments and in the control groups. Due to similar sinking velocities and hence similar volume specific mass of treated and untreated daphnids, it is not possible to distinguish the energy expenditure they had to spend against gravity, i.e. with or without accumulated nanoparticles on their exoskeleton.

### Preferred residence and swarming behavior

**Figure 2:**
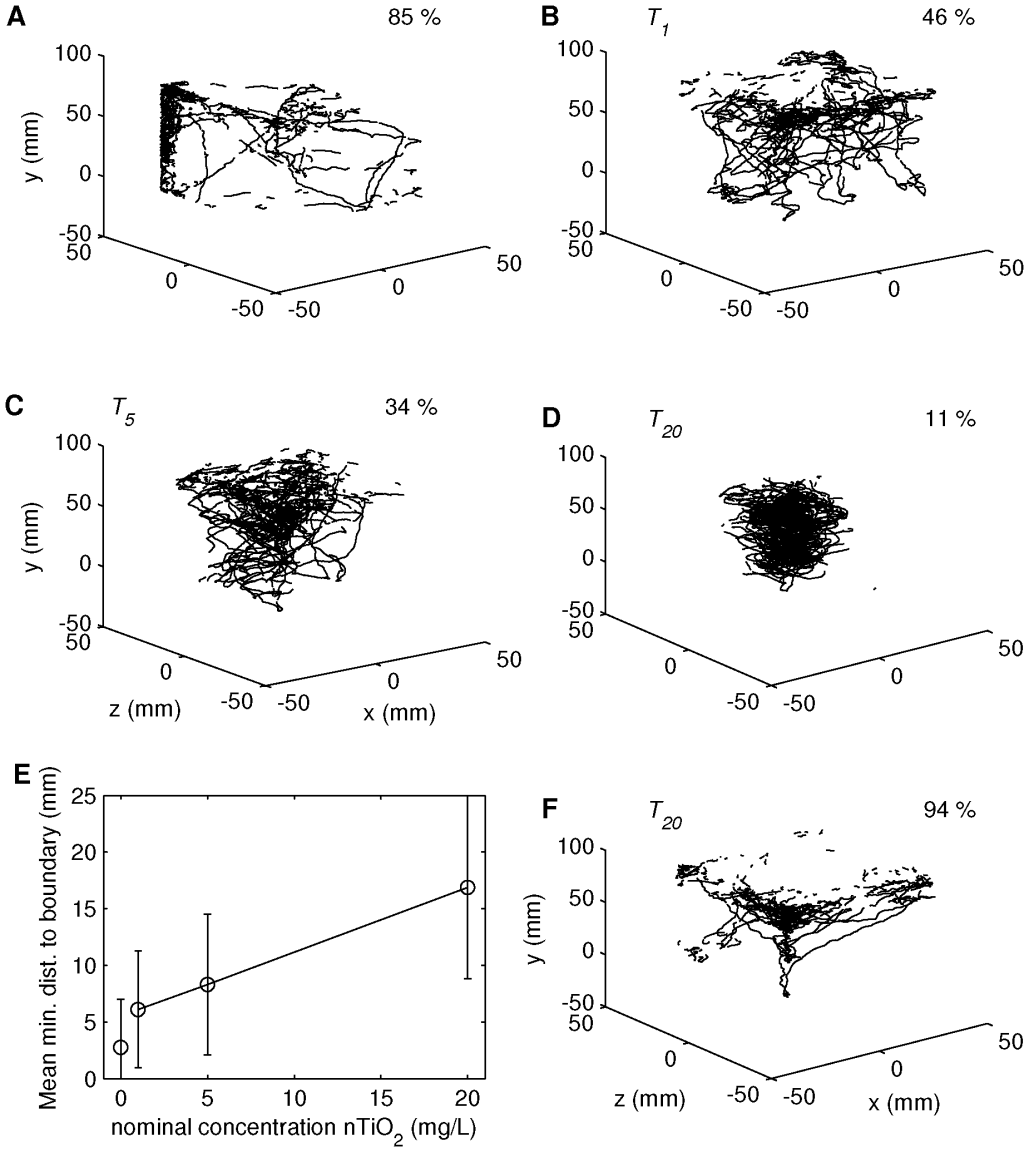
Exemplary swimming trajectories. Simultaneously moving daphnids during measurements of (A) *C*, (B) *T*_1_, (C) *T*_5_ and (D) *T*_20_ at *t*_0_, i.e., directly after application and E group-wise averaged minimal distance to a solid boundary at *t*_0_ (circles and solid line connecting the distances of the treatment measurements, error bars denote standard deviations). (F) Swimming trajectories of *T*_20_ at *t*_24_. Values in the upper right corners of subplots (A-D) and (F) denote the percentage of residence in vicinity (≤ 5 mm) to the boundaries of the experimental tank.

The three-dimensional pathways in *C* and *T*_1,5,20_ directly after application of nTiO_2_ (Figure 2A-D) suggest an increasing tendency for aggregation of the daphnids in the center of the test vessel, i.e. swarming behavior, for increasing particle concentrations. Figure 2E shows mean minimum distances between daphnids and the vessel boundary as a function of particle concentration for *t*_0_. The mean minimum distance corresponds to the mean value of minima of perpendicular distances between each individual and the boundaries of the cubic vessel. While the mean minimum distance was < 3 mm in the control *C*, it was consistently > 6 mm in the treatments and increased consistently with particle concentration. At *t*_24_, the preferred swimming in the central region of the test vessel was not present anymore, even for *T*_20_ (Figure 2F). Overall, 68 % of all daphnids were detected in vicinity (≤ 5 mm) of the vessel boundaries. Please note that at *t*_0_ nTiO_2_ was homogenously suspended and hence turbidity and therewith diffusive light intensity can be expected to be highest. In contrast at *t* ≥ *t*_24_ nearly 50 % of the nTiO_2_ particles were agglomerated and deposited at the bottom of the vessel. Avoidance of the bottom layer of agglomerated nTiO_2_ deposits could not be observed in treatments *T*_1,5,20_ in comparison to the control *C*. The residence close to the bottom boundary was 3±3 % during all observations, irrespective of treatment or control groups.

### Swimming velocity

**Figure 3:**
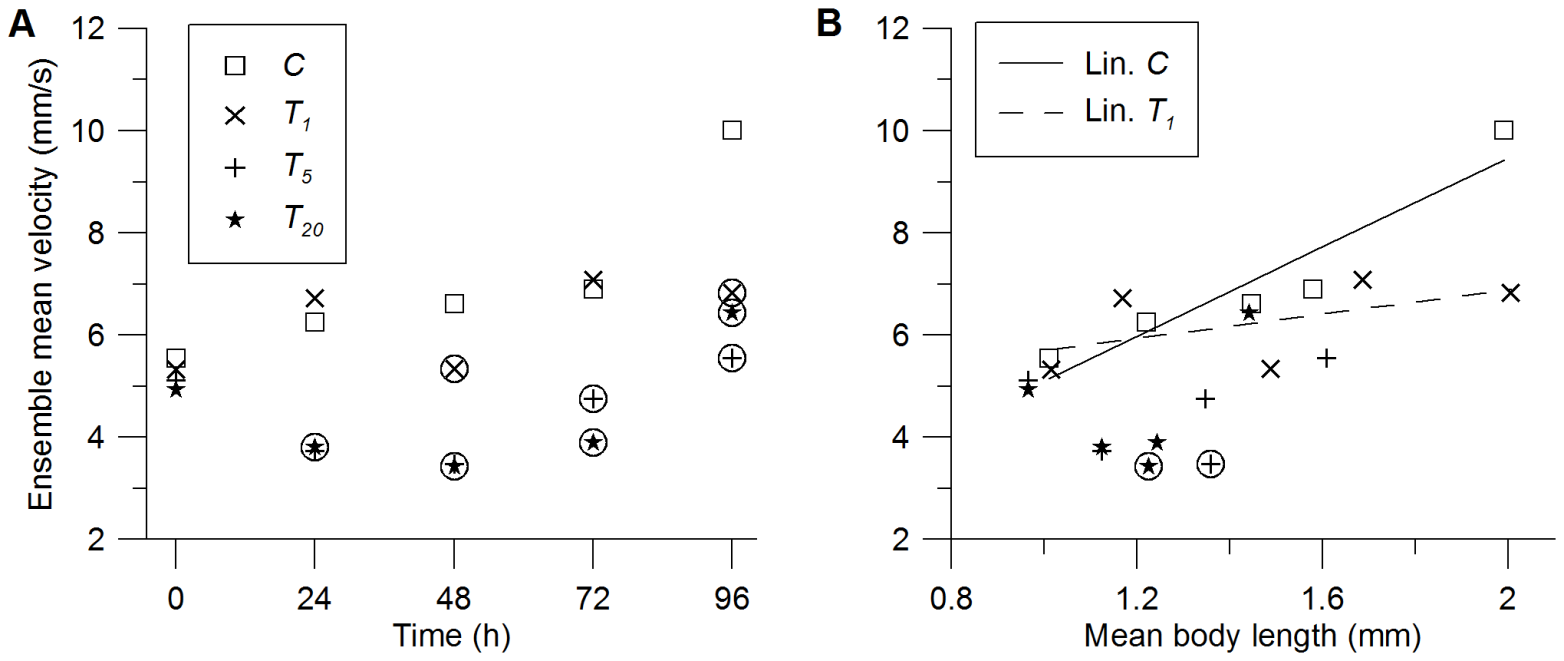
Ensemble mean swimming velocities. 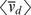
 of *Daphnia magna* (A) at times after application and (B) in relation to corresponding body lengths. Error bars are omitted for the sake of clarity. Please refer to Table S2 for standard deviations. Velocities significantly different (*p*<0.05) from the control are surrounded by circles. Please note that only velocities of similar body lengths (1.22 mm for *C*, 1.44 mm for *T*_5_ and 1.23 mm for *T*_20_) had been tested with respect to significant differences. Linear regression for *C* with d*v*/d*L* = 4.4 1/s (black line, *r* ^2^ = 0.87) and for *T*_1_ with d*v*/d*L* = 1.2 1/s (grey line, *r* ^2^ = 0.3).

Ensemble-mean swimming velocity 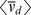 (Figure 3A) of all groups was almost similar (5.2±0.2 mm/s) at *t*_0_, i.e. directly after application of nTiO_2_. However, they varied between 3.7 mm/s (*T*_5_) and 6.7 mm/s (*T*_1_) at *t*_24_. Although ensemble-mean velocities are widely scattered at all *t* > *t*_0_, it is evident that swimming velocities of *T*_5, 20_ were always below those of *C* and *T*_1_. Differences between *C* and *T*_5, 20_ were significant (Figure 3A; Table S2) for all *t* > *t*_0_, in *T*_1_ daphnids swam significantly slower at *t*_48_ and *t_96_* than those in *C*.

Differences in swimming behaviors can potentially be caused by smaller body lengths resulting from reduced growth rates [17]. However, even size-independent swimming velocities (Figure 3B) denote a clear difference between ensemble-mean velocities of *C*, *T*_1_ and ensemble-mean velocities of *T*_5,20_ for body lengths between 1.1 and 1.4 mm. Within this range one has to compare velocities of one record (*t*­_24_) of *C* and *T*_1_ with three records (*t*_24, 48, 72_) of *T*_5,20_ due to the considerably slower growth in *T*_5_ and *T*_20_. Even smaller sized daphnids in *C* (*L* = 1.22 mm, *t*_24_) swam significantly faster than the larger daphnids of *T*_5_ (*L* = 1.36 mm, *t*_48_, *p* = 0.003) and *T*_20_ (*L* = 1.23 mm, *t*_48_, *p* = 0.009). At *t*_96_, we observed a significantly smaller swimming velocity for *T*_1_ compared to the control (Figure 3B), although the animal size was with 2.0±0.15 mm and 2.0±0.16 mm virtually identical. The slope of the size-dependent swimming velocities for the linear regression 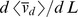 (Figure 3B) was significantly larger in *C* than in *T*_1_ (*p* = 0.046) indicating a size-independent reduction of the swimming velocity even in *T*_1_.

**Figure 4:**
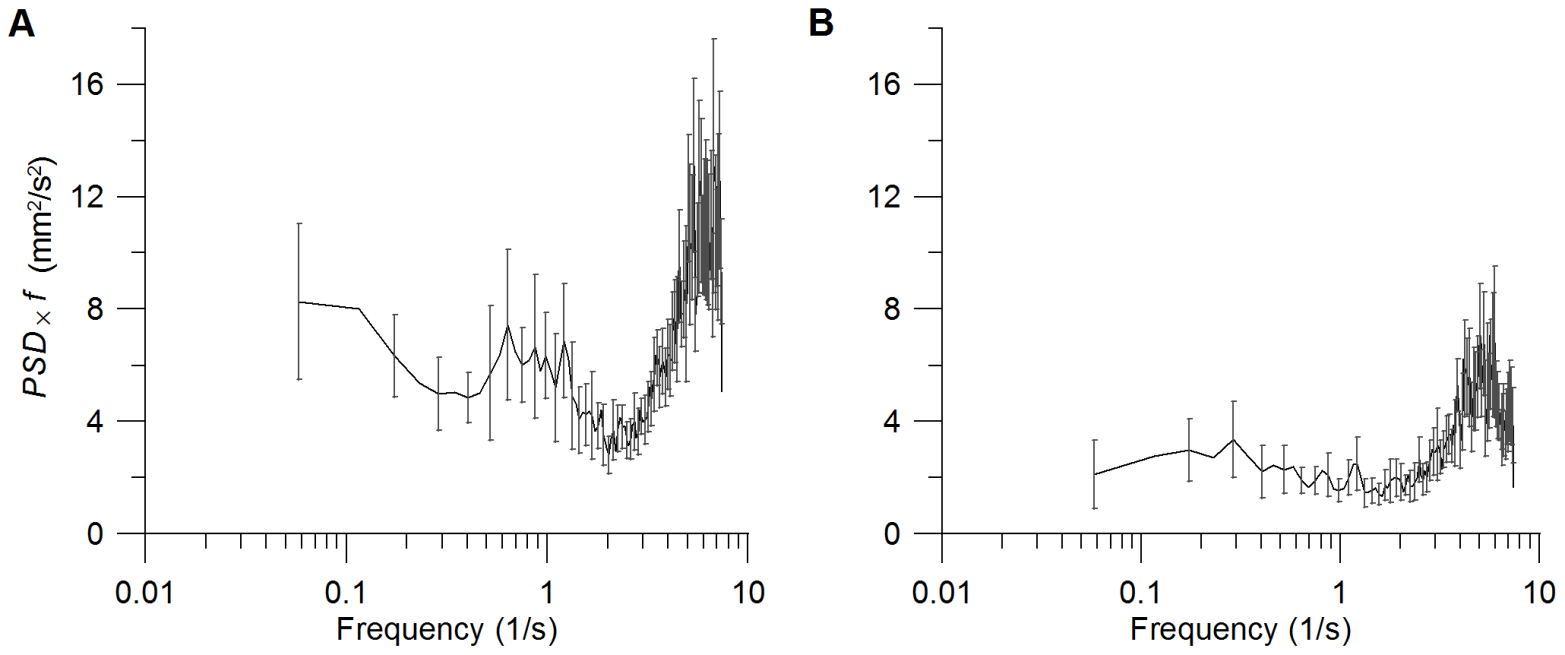
Variance preserving power spectral density. Presentation of *PSD ^.^ f* of the vertical swimming velocity component of (A) *C* and (B) *T*_5_ at *t*_96_. Grey error bars show the standard deviation of individual velocity spectra.

*PSD* of swimming velocities show a persistent spectral gap between *f* = 1 Hz and 2.5 Hz (Figure 4), separating two spectral ranges of elevated velocity variance associated with higher and lower frequencies, respectively. While low-frequency (*f*<1 Hz) variance is induced by larger scale motions, like e.g. cruising across the entire vessel, high-frequency (*f* > 1 Hz) velocity variance can be associated with antenna motions with typical beat frequencies between of 3–5 Hz [17]. Due to limited record lengths of organism pathways and therewith increased statistical uncertainty we disregarded the low frequency range of PSD, i.e. we do not consider potential differences in velocity variance at larger scale motions. The maximum spectral variance in the high-frequency range of the spectra is significantly smaller in *T*_5, 20_ in comparison to *C* (Table S3). However, differences were not significant between *C* and *T*_20_ at *t*_0_ and between *C* and *T*_5_ at *t*_48_ (Table S3). Also the integral variance 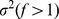 of *C* was significantly higher in comparison to corresponding values of *T*_5_ and *T*_20_, except for *T*_5_ at *t*_48_. In contrast to the maximum spectral variance, 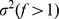 also differs significantly at *t*_0_ and also between *C* and *T*_1_ at *t*_0_ and *t*_72_. Although our records did not allow for a direct analysis of individual antenna strokes the spectral analysis of the high-frequency range clearly show that *T*_5_ and *T*_20_ motions contain less kinetic energy and hence organisms showed reduced swimming activity.

## Discussion

### Immobilization and growth

Less than 20 % of the test organisms were immobilized during our experiments, suggesting that acute lethal or other stronger toxicity effects were not of importance during the tests. This finding does not contradict observations by Dabrunz et al. [13], who observed immobilization (96-h EC_50_ = 0.73 mg/L) of juvenile daphnids exposed to A-100 nTiO_2_ in ISO medium, since we used older daphnids and a different type of both medium and nTiO_2_ particles in the present study.

Growth was monitored during the present study to account for differences in body size, when analyzing the swimming performance data. Based on these data and the information available from the literature [12], the growth of *D. magna* in a 96 h test appears to be a reasonable endpoint. Dabrunz et al. [13] suggested to prolong the acute test duration for *D. magna* and nTiO_2_ to 96 h, since this time period allows for the specific mechanisms of toxicity to occur. We found a reduced growth within the observation period in the two highest treatments of 5 and 20 mg/L nTiO_2_. This is to the best of our knowledge the first study reporting a negative effect of a 96 h nTiO_2_ exposure on the growth of *D. magna*. Although body length and growth rates depend on multiple abiotic and biotic factors, e.g. on temperature and kairomone (message chemical) concentration [8], one of the most important factor is the feeding rate [31]. In the present experiment, however, the daphnids were not fed at all during the 96-h experimental phase. Despite this lack of additional food provision, measured growth rates during this experiment corresponded to growth rates measured by Gliwicz [32] in *Daphnia* held at maximal food concentrations. It is likely, though we cannot provide proof based on the present data, that the observed reduced growth is related to delayed or disrupted molting of daphnids exposed to nTiO_2_, an effect which has already been described in *D. magna* following exposure to 2 mg/L of the nTiO_2_ product A-100 [13]. A comparison of the estimated body lengths with data of Baillieul and Blust [17], who investigated the impact of Cd^2+^, shows that daphnids in the current experiment were always approximately 0.5 mm smaller. But similar to our observations, differences in body lengths appeared at *t* > *t*_48_ following exposure to Cd^2+^. The biological surface coating of nTiO_2_ particles on the daphnids exoskeleton [13], which we observed also in the present experiments, did not translate into appreciable differences in measured size-related sinking velocities and corresponding mass densities.

### Impact of nTiO_2_ on preferred residence

The observed predominant residence of all groups in the vicinity to the test vessel boundaries might be explained by the well-known behavior of *Daphnia* grazing algae growing on submerged surfaces [33]. The test organisms showed during the initial phase of the experiment (*t*_0_) a clearly concentration-dependent organized and circular swimming pattern in the central part of the vessel. This may be interpreted as a swarming behavior which has already been observed in various *Daphnia* species as a response to cues from food, competitors or predator [34]. An experimental study on *Daphnia pulicaria* by Szulkin et al. [35], suggested that uniformity in swimming patterns is based on individual perception of both kairomones and light in the environment rather than on social interactions. Although we are not aware of the underlying mechanism, the swarming behavior which has been regularly observed in *Daphnia* as a response to predator-induced stress, or vice-versa as a response to attractors, also seems to be triggered by the presence of nTiO_2_. The measured concentration decline of nTiO_2_ in the water column (Figure S1), which is well in accordance with results of Dabrunz et al. [13] for A-100 nTiO_2_ in ISO medium and with observations made in Seitz et al. [7], clearly indicates the formation of a bottom layer. Both Seitz et al. [7] and Bundschuh et al. [36] have shown for P25 nTiO_2_ this bottom layer to be of crucial importance in the exposure pathway. The formation of the bottom layer has been measured here via a decrease in available Ti in the water column, as it was also done in previous studies. The formation of the bottom layer, however, can easily be verified by visual inspection accommodating the Ti analysis. We also reported the polydispersity indices (PI) of our particle size measurements and noted that the dynamic light scattering measurements led to too high PI values already about 2 hours following the start of a test. This suggests the formation of larger aggregates of about 1400 nm to occur relatively fast. However, Seitz et al. [7] amongst others also suggested that daphnids may ingest larger nanoparticle agglomerates even at higher rates and thus be affected negatively by the uptake. Interestingly, even the presence of this bottom layer of agglomerated nTiO_2_ as observed in the present study did not lead to a reduced presence of daphnids in the vicinity of the bottom compared to the control setup. As *D. magna* is known to feed from the bottom [37], it can be hypothesized that the specimens even increase their nTiO_2_-exposure as it has been shown in other recent studies for *D. magna* [7] and the amphipod *Gammarus fossarum* [36].

### Swimming velocity

Our results show an impact of individual size on the ensemble mean swimming velocity, a phenomenon already observed in studies with other chemical stressors [17]. Notably, also the swimming velocities of similar sized daphnids were reduced already for nTiO_2_-exposure as low as 1 mg/L. This suggests that nTiO_2_ indeed affect the mechanical ability of locomotion negatively and that this is not only an indirect effect of the reduced growth. This kind of effect has up to now not been shown as a consequence of nanoparticle exposure. Reduction of swimming velocity was between 25 and 40 % in comparison to levels in the control at *t*_96_. However, the limited number of treatments prevented the determination of a no-effect threshold.

The estimated mean velocities in the present study were approximately two-fold higher in comparison to values of Baillieul and Blust [17] and 1.5-fold in comparison to Duquesne and Küster [22] or Lechelt et al. [38]. However, this discrepancy might be related to the two-dimensional observations made during all those studies which may have underestimated velocities without compensation for the unsolved third velocity component. We noticed that 
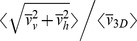
 =  0.77 (with *v*_h1_) and 0.78 (with *v*_h2_), i.e. that both mean values of horizontal velocity components are equal and a factor of 1.5 compensate the missing horizontal velocity in two-dimensional observations for mean swimming velocity estimations. Swimming velocities of similar magnitude like in our observations were estimated by Dodson et al. [15] who also measured three-dimensional *Daphnia* swimming velocities.

Duquesne and Küster [22] observed that only changes of the mean swimming velocity indicate a significant change in swimming behavior following an exposure to a toxic substance. Other parameters, like speed distribution and fractal dimension did not change. In the present study, we found that also the high-frequency variance of swimming velocity was significantly affected by higher concentration of nTiO_2_. Maximum spectral power at beat frequencies typical for the daphnids 2^nd^ antennae reflect the ‘hop and sink’ swimming mode [24]. The fact that *PSD* was reduced verifies that exposed daphnids were less active. The analysis of spectral distributions of velocity variance was – to the best of our knowledge – applied for the first time for investigating the impact of toxic substances on zooplankton swimming behavior. It is likely that the biological surface coating by nTiO_2_, which in previous studies had been shown to be particularly affect the antennae [13], is the cause for the observed decrease in swimming activity. During another study [7] we used P25, ASTM medium and *Daphnia magna* and thus exactly the same conditions as used in the present manuscript. We observed in that study, that no coating was present when the medium was amended with dissolved organic carbon (DOC), and we concluded that due to the presence of DOC the nTiO_2_ remained in the water column as smaller sized particles. In the present study we did not amend the test medium with DOC, therefore, we again observed a biological surface coating visible as TiO_2_-deposits on the daphnids surface (Figure S2 in the Supporting Information).

Spectral analysis of swimming behavior can be expected to be a powerful tool for investigating the mechanical impacts of sub-lethal concentrations of toxicants on motile aquatic organisms, when mean swimming velocity and its variance do not provide sufficient insights into exposure effects. In the present study, coating of the exoskeleton of daphnids by nTiO_2_ likely reduced the mobility of the antennae, inhibited or delayed molting or may have been ingested and thus reduced an efficient metabolism. The observed decrease in swimming activity is in accordance with previous findings that daphnids show even slightly increased lipid contents following short-term (48 h) exposure to nTiO_2_ [13].

Lovern et al. [21] did, in contrast to the present study, not observe any behavioral changes of *D. magna* following exposure to 2 mg/L nTiO_2_. The most likely explanation is that the observation period in Lovern et al. [21] was restricted to 2.5 h.

### Ecological implication

Although the initial nTiO_2_ concentrations in our experiments were 1, 5 and 20 mg/L, the effective concentration of suspended nanosized particles decreased strongly during the present experiment. Dabrunz et al. [13] also observed that due to formation of agglomerates and their deposition, the concentration of nTiO_2_ in suspension decreased strongly with time (e.g. maximal concentration from 8 mg/L at *t*_0_ to 1.06 mg/L at *t*­_24_). However, the current study as well as the experiment of Dabrunz et al. [13] had been conducted in vessels without turbulence or even weak shear velocity which might not be directly comparable to natural surface waters. There, nTiO_2_ particles may not deposit so fast or even faster if agglomeration is accelerated by increasing collision frequency [39]. Furthermore, the presence of dissolved organic carbon may reduce the tendency of nTiO_2_ to form biological surface coating [7].

The lowest levels of concentrations leading to acute sub-lethal effects in our observations (1 mg/L) are low in comparison to those observed in many other acute [10] and even long-term studies [40]. They are still at least four orders of magnitude above levels of about 2^.^10^-4 ^mg/L, which are predicted to occur in surface waters [5]. However, other recent studies using longer exposure periods showed that exposure levels as low as 0.02 mg/L nTiO_2_ alter the sensitivity of offspring born from previously exposed parental *D. magna* [41].

Ecological implications of the analyzed size and behavioral responses of *D. magna* may be manifold. Because of *Daphnia’*s key role in freshwater aquatic food webs, various predator-prey interactions are potentially affected. A reduced growth rate and reduced swimming speed of *D. magna* may increase predation by size-dependent invertebrate predators [8]. Furthermore, due to a reduced mean velocity as well as due to reduced swimming variance, nTiO_2_ affected daphnids may show a reduced avoidance of predation by fish.

The results of the present study highlight the potential of the swimming behavior analysis by video recording to analyze specifically nanoparticle effects on zooplankton organism. A variety of additional endpoints become available in order to quantify sub-lethal effects of nanoparticles on motile aquatic organisms as part of their risk assessment.

## Supporting Information

Figure S1 **Concentration of nTiO_2_** **suspended in water over a 96-h time period**. Initial concentration was 2.5 mg TiO_2_/L (P25, Evonik Aeroxide®). Measured as ^49^Ti (mean ± standard deviation; n = 5) in ASTM (American Society of Testing and Materials) reconstituted hard fresh water (192 mg/L NaHCO_3_, 120 mg/L CaSO_4_·2H_2_O, 120 mg/L MgSO_4_, 8 mg/LKCl).
(DOC)

Figure S2 **Biological surface coating by nTiO_2_ on** ***Daphnia magna*** **.** Daphnids were exposed for 24 hours to 2 mg/L P25 TiO_2_ in ASTM medium (Photograph: Frank Seitz).
(DOC)

Table S1 **Mean body length ± standard deviation [mm] at sampling times after application.**
(DOC)

Table S2
**Ensemble-mean velocity** 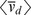 **± standard deviation [mm/s] at sampling times after application.**
(DOC)

Table S3
**Maximum spectral variance (** 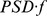 **) and integrated high-frequency variance (** ***f*** **> 1 Hz) (** 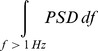 **) (shown in parentheses) in [mm^2^/s^2^] at sampling times after application.**
(DOC)
