# Supplementary material for: Three-Dimensional Analysis of the Swimming Behavior of Daphnia magna Exposed to Nanosized Titanium Dioxide
Source: PLoS One. 2013 Nov 18;8(11):e80960. doi: 10.1371/journal.pone.0080960 (PMC3832431; doi:10.1371/journal.pone.0080960)
Supplement: Figure S1 — Concentration of nTiO2 suspended in water over a 96-h time period. Initial concentration was 2.5 mg TiO2/L (P25, Evonik Aeroxide®). Measured as 49Ti (mean ± standard deviation; n = 5) in ASTM (American Society of Testing and Materials) reconstituted hard fresh water (192 mg/L NaHCO3, 120 mg/L CaSO4·2H2O, 120 mg/L MgSO4, 8 mg/LKCl). (DOC) [file pone.0080960.s001.doc]

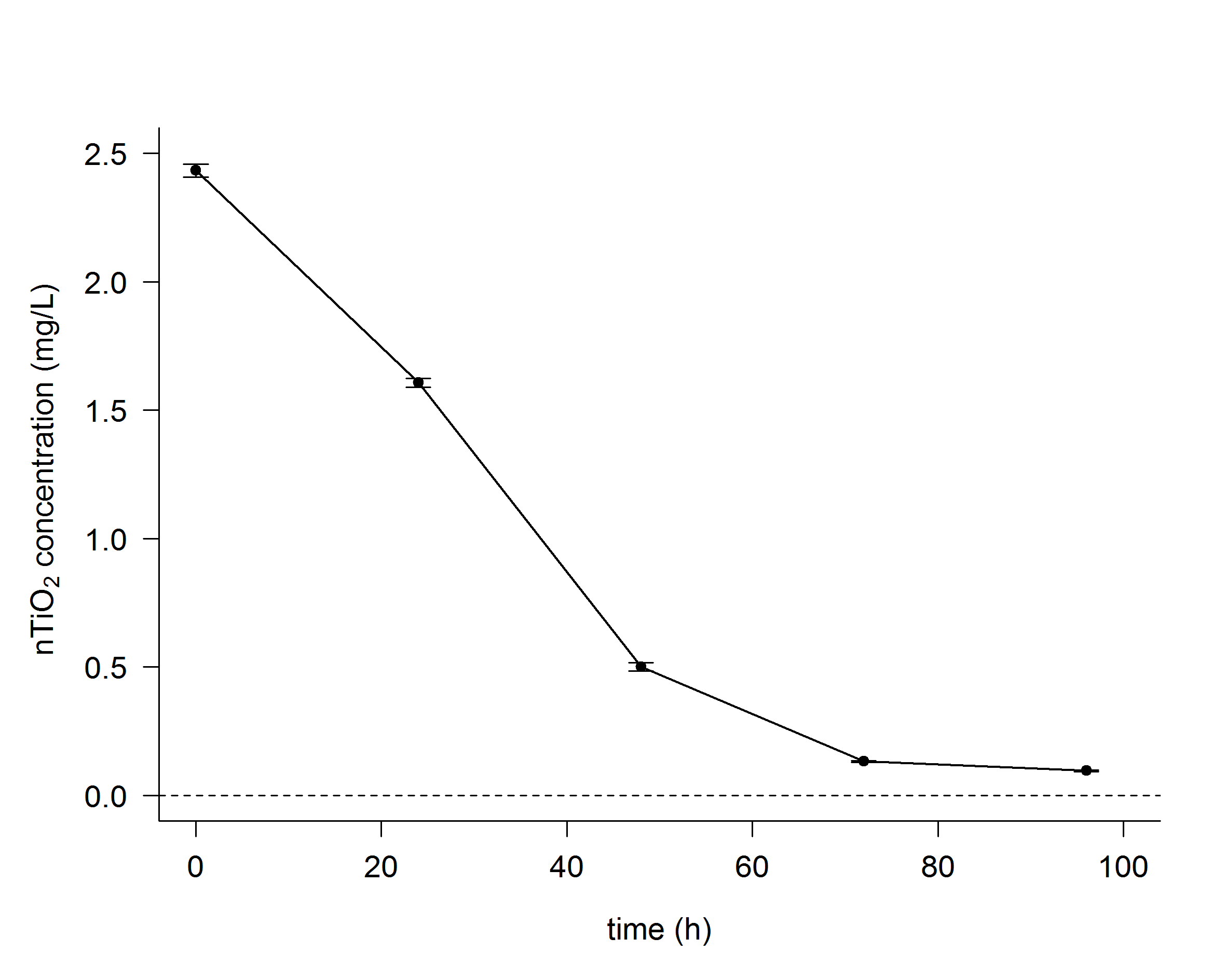


**Figure S1. Concentration of nTiO_2_** **suspended in water over a 96-h time period**. Initial concentration was 2.5 mg TiO_2_/L (P25, Evonik Aeroxide®). Measured as ^49^Ti (mean ± standard deviation; n=5) in ASTM (American Society of Testing and Materials) reconstituted hard fresh water (192 mg/L NaHCO_3_, 120 mg/L CaSO_4_·2H_2_O, 120 mg/L MgSO_4_, 8 mg/L KCl).
