# Supplementary material for: Three-Dimensional Analysis of the Swimming Behavior of Daphnia magna Exposed to Nanosized Titanium Dioxide
Source: PLoS One. 2013 Nov 18;8(11):e80960. doi: 10.1371/journal.pone.0080960 (PMC3832431; doi:10.1371/journal.pone.0080960)
Supplement: Figure S2 — Biological surface coating by nTiO2 on Daphnia magna . Daphnids were exposed for 24 hours to 2 mg/L P25 TiO2 in ASTM medium (Photograph: Frank Seitz). (DOC) [file pone.0080960.s002.doc]

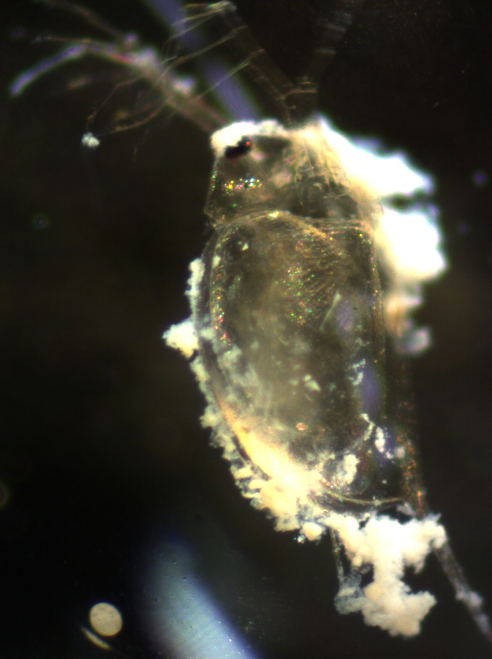


**Figure S2. Biological surface coating by nTiO_2_ on *Daphnia magna*.** Daphnids were exposed for 24 hours to 2 mg/L P25 TiO_2_ in ASTM medium (Photograph: Frank Seitz).
