# Supplementary material for: Three-Dimensional Analysis of the Swimming Behavior of Daphnia magna Exposed to Nanosized Titanium Dioxide
Source: PLoS One. 2013 Nov 18;8(11):e80960. doi: 10.1371/journal.pone.0080960 (PMC3832431; doi:10.1371/journal.pone.0080960)
Supplement: Table S1 — Mean body length ± standard deviation [mm] at sampling times after application. (DOC) [file pone.0080960.s003.doc]

**Table S1.** Mean body length ± standard deviation [mm] at sampling times after application.

| **Group** | ***t_0_*** | ***t_24_*** | ***t_48_*** | ***t_72_*** | ***t_96_*** |
| --- | --- | --- | --- | --- | --- |
| *C* | 1.01±0.05 | 1.22±0.10 | 1.45±0.09 | 1.58±0.11 | 1.99±0.15 |
| *T_1_* | 1.02±0.09 | 1.17±0.09 | 1.49±0.11 | 1.69±0.13 | 2.01±0.16 |
| *T_5_* | 0.96±0.09 | 1.12±0.08 | 1.36±0.07*^0^ | 1.35±0.11** | 1.61±0.26** |
| *T_20_* | 0.96±0.05 | 1.12±0.13 | 1.23±0.07** | 1.24±0.09** | 1.44±0.29** |
| *C** | 1.02±0.05 | 1.26±0.06 | 1.54±0.15 | 1.74±0.10 | 2.06±0.08 |

* significant differences to *C* at levels *p* < 0.05

** significant differences to *C* at levels *p* < 0.01

^0^ failures due to Bonferoni correction
