# Supplementary material for: Three-Dimensional Analysis of the Swimming Behavior of Daphnia magna Exposed to Nanosized Titanium Dioxide
Source: PLoS One. 2013 Nov 18;8(11):e80960. doi: 10.1371/journal.pone.0080960 (PMC3832431; doi:10.1371/journal.pone.0080960)
Supplement: Table S2 — Ensemble-mean velocity ± standard deviation [mm/s] at sampling times after application. (DOC) [file pone.0080960.s004.doc]

**Table S2.** Ensemble-mean velocity $\left\langle\overline{v}_{d}\right\rangle$± standard deviation [mm/s] at sampling times after application.

| **Group** | ***t_0_*** | ***t_24_*** | ***t_48_*** | ***t_72_*** | ***t_96_*** |
| --- | --- | --- | --- | --- | --- |
| *C* | 5.54±0.92 | 6.26±0.80 | 6.61±0.53 | 6.91±0.65 | 10.01±0.68 |
| *T_1_* | 5.33±0.44 | 6.73±0.72 | 5.33±1.17*^0^ | 7.09±1.06 | 6.83±1.22**^0^ |
| *T_5_* | 5.12±0.53 | 3.73±1.17** | 3.48±1.20** | 4.76±1.79* | 5.55±1.05** |
| *T_20_* | 4.94±0.65 | 3.82±1.06** | 3.44±0.96** | 3.90±1.56**^0^ | 6.44±0.96** |

* significant differences to *C* at levels *p* < 0.05

** significant differences to *C* at levels *p* < 0.01

^0^ failures due to Bonferoni correction
