# Supplementary material for: Three-Dimensional Analysis of the Swimming Behavior of Daphnia magna Exposed to Nanosized Titanium Dioxide
Source: PLoS One. 2013 Nov 18;8(11):e80960. doi: 10.1371/journal.pone.0080960 (PMC3832431; doi:10.1371/journal.pone.0080960)
Supplement: Table S3 — Maximum spectral variance ( ) and integrated high-frequency variance ( f > 1 Hz) ( ) (shown in parentheses) in [mm2/s2] at sampling times after application. (DOC) [file pone.0080960.s005.doc]

**Table S3.** Maximum spectral variance ($PSD\cdot f$) and integrated high-frequency variance (*f* > 1 Hz) ($\int_{f > 1 Hz} PSD df$) (shown in parentheses) in [mm^2^/s^2^] at sampling times after application.

| **Group** | ***t_0_*** | | ***t_24_*** | | ***t_48_*** | | ***t_72_*** | | ***t_96_*** | |
| --- | --- | --- | --- | --- | --- | --- | --- | --- | --- | --- |
| *C* | 9.84 | (9.14) | 14.67 | (10.27) | 9.57 | (7.62) | 14.68 | (12.31) | 13.09 | (11.57) |
| *T_1_* | 6.94 | (5.59**) | 15.16 | (11.15) | 9.68 | (7.93) | 12.43 | (8.80) | 17.77 | (9.03) |
| *T_5_* | 6.77*^0^ | (5.64**) | 6.67** | (6.39**^0^) | 7.69 | (5.89) | 8.53* | (6.70**) | 8.34*^0^ | (6.70**) |
| *T_20_* | 7.93 | (6.32**) | 7.82*^0^ | (6.94**) | 6.53*^0^ | (5.46*^0^) | 7.93** | (6.19**) | 7.82**^0^ | (6.74**^0^) |

* significant differences to *C* at levels *p* < 0.05

** significant differences to *C* at levels *p* < 0.01

^0^ failures due to Bonferoni correction
